# Radiation therapy and vaccination against tumor-specific EGFRvIII effectively clears tumors in a murine model of head and neck squamous cell carcinoma

**DOI:** 10.1186/2051-1426-3-S2-P78

**Published:** 2015-11-04

**Authors:** Lauren Uhde, Shelly Bambina, Alejandro Alice, Peter Lauer, Marka Crittenden, Keith S Bahjat, Michael Gough

**Affiliations:** 1 Earle A. Chiles Research Institute, Portland, OR, USA; 2 Molecular Microbiology and Immunology, OHSU, Portland, OR, USA; 3 Aduro Biotech, Berkeley, CA, USA; 4 Providence Cancer Center; The Oregon Clinic, Portland, OR, USA; 5 The Oregon Clinic, Portland, OR, USA; 6 Robert W. Franz Cancer Research Center, Portland, OR, USA

## 

EGFRvIII is a constitutively active and tumor-specific deletional mutant of EGFR found in multiple tumor types including glioblastoma multiforme, and has been reported in head and neck squamous cell carcinomas (HNSCC). The deletion of *EGFR* exons 2-7 results in a novel glycine at the junction and yields a tumor-specific antigen with demonstrated immunogenicity in mice and humans. Using a live-attenuated *Listeria monocytogenes*- based vaccine expressing a 21-AA neo-peptide from EGFRvIII (LmEGFRvIII), we demonstrated that prophylactic vaccination protects against subsequent challenge with an EGFRvIII-expressing squamous cell carcinoma (SCCVII-EGFRvIII). Similarly, therapeutic vaccination three days post-implantation prevented outgrowth of EGFRvIII-expressing tumors but not parental EGFRvIII-negative tumors. Conversely, we found that LmEGFRvIII vaccination was insufficient to clear large established SCCVII-EGFRvIII tumors despite eliciting large numbers of polyfunctional EGFRvIII-specific CD8+ T cells. We hypothesized that localized inflammation elicited by radiation therapy could recruit EGFRvIII-specific CD8+ T cells into the tumor. We demonstrated that while neither LmEGFRvIII vaccination nor radiation therapy alone were able to control the EGFRvIII-expressing tumors, the combination of LmEGFRvIII and radiation therapy led to tumor regression and durable cures. We are currently exploring the potential mechanisms for their synergy including the role of T cell recruitment, survival and epitope spreading.

